# Association between serum free fatty acids and gestational diabetes mellitus

**DOI:** 10.3389/fendo.2024.1451769

**Published:** 2024-11-28

**Authors:** Danyang Li, Haoyi Du, Na Wu

**Affiliations:** ^1^ Department of Endocrinology, Shengjing Hospital of China Medical University, Shenyang, China; ^2^ Department of Pediatrics, Shengjing Hospital of China Medical University, Shenyang, China

**Keywords:** gestational diabetes mellitus, free fatty acid profile, adverse pregnancy outcomes, liquid chromatography-mass spectrometry, C18:0

## Abstract

**Background:**

Pregnant women with gestational diabetes mellitus (GDM) are at an increased risk of adverse pregnancy outcomes (APO). Early understanding of risk factors affecting these outcomes may facilitate preventive interventions for women at high risk. Blood samples from GDM and control pregnant women were collected for Free fatty acid (FFA) profiling to determine the relationship with the occurrence of APO in GDM pregnant women.

**Methods:**

The study comprised 144 women diagnosed with GDM and 52 normal control pregnancy (NC). Venous fasting serum samples were collected during the second trimester. The serum FFA levels were detected by liquid chromatography-mass spectrometry (LC-MS). The primary outcome consisted of serious maternal and neonatal adverse events ( hypertensive disorder complicating pregnancy (HDCP), emergency cesarean section, large for gestational age (LGA), small for gestational age (SGA), macrosomia, low birth weight (LBW), preterm birth, and stillbirth). The association of metrics with outcomes was assessed, and receiver operating characteristic (ROC) curve analysis was employed to evaluate clinical utility.

**Results:**

Differences in fatty acid profiles were observed between GDM patients and controls. Stearic acid (C18:0) levels differed between the normal pregnancy outcome (NPO) and APO groups, potentially correlating with fetal sex. Logistic regression models indicated that moderate and high levels of C18:0 were negatively associated with APO relative to the NPO group. ROC analysis demonstrated that C18:0 had a certain predictive ability for APO, and predictive efficiency was enhanced when combined with general clinical data.

**Conclusion:**

The level of C18:0 was associated with the occurrence of APO in pregnant women with GDM and exhibited a certain predictive value. When C18:0 was combined with general clinical data, the predictive power for APO was improved.

## 1 Introduction

Gestational diabetes mellitus (GDM) refers to abnormal glucose tolerance that occurs or is first detected during pregnancy (1). GDM is one of the most common pregnancy complications. Its prevalence has increased by >35% in recent decades and continues to grow (2). GDM is characterized by insufficient relative insulin secretion (3), which cannot compensate for the gradual increase of insulin resistance (IR) during pregnancy (4), leading to maternal hyperglycemia. During pregnancy, the release of placental hormones promotes IR, increases lipolysis, and elevates maternal plasma-free fatty acid (FFA) levels, thereby inhibiting maternal glucose uptake and stimulating hepatic gluconeogenesis (5, 6). Lipids can lead to the development of GDM by affecting IR. A high-fat, high-sugar diet leads to IR and β-cell dysfunction (7). Lipotoxicity caused by hypertriglyceridemia during pregnancy also leads to pancreatic β-cell damage, further reducing insulin secretion (8, 9). Studies have shown that palmitic acid, stearic acid, arachidonic acid (AA), dihomo-γ-linolenic acid (DGLA), and docosahexaenoic acid (DHA) positively correlated with higher homeostatic model assessment of IR(HOMA-IR) and C-peptide. This indicates that palmitic acid, stearic acid, AA, DGLA, and DHA may affect GDM development (10).

FFAs, also known as non-esterified fatty acids, are hydrocarbon chains composed of a methyl group at one end and a carboxyl group at the other. Depending on the number of carbon-carbon double bonds, FFA can be divided into saturated fatty acids (SFAs) (without double bonds), monounsaturated fatty acids (MUFAs) (one double bond), and polyunsaturated fatty acids (PUFAs) (multiple double bonds). FFA, physiologically important energy substrates, are released from adipose tissue through lipolysis according to the body's energy demand. The levels of most FFAs gradually decrease from the first to third trimester (11). Therefore, FFA levels may differ among pregnant women (12). FFA profiles encompass various types of FFA, and specific FFAs are associated with the risk of GDM (11, 13).

Pregnant women with GDM can experience complications with various adverse pregnancy outcomes (APO), such as preterm birth, cesarean section, macrosomia, preeclampsia, low birth weight (LBW), and intrauterine growth retardation (14–16). Moreover, GDM is associated with long-term childhood obesity and abnormal glucose tolerance (17, 18). Early understanding of the factors influencing adverse outcomes and prevention is crucial. Studies have shown that changes in lipid levels can lead to adverse maternal and infant outcomes. Maternal triglyceride (TG) levels may be associated with fetal birth weight (19). FFAs obtained from the breakdown of TGs in the body are associated with preterm birth, preeclampsia, and fetal birth weight (20–23). In pregnant women with GDM, high maternal FFA levels may lead to high fetal birth weights and macrosomia (24–26). However, high FFA levels in pregnant women with GDM in the third trimester are related to the occurrence of fetal growth restriction (FGR) (27). Additionally, the effect of GDM on FFA levels can be profound, leading to differences in FFA profile levels in postpartum women with a GDM history (28). In addition, during the follow-up of women with GDM within 5 years after delivery, the metabolome, including linoleic acid, was associated with abnormal glucose metabolism after delivery (29).

In summary, the FFA level/spectrum is related to IR and may be involved in the development of GDM. FFA levels alone may be associated with adverse outcomes in women with GDM. Few studies have correlated the changes in FFA spectrum levels in patients with GDM and adverse maternal or neonatal outcomes. GDM is related to various APOs and increases the risk of long-term maternal and infant complications. Most studies focused on analyzing the general clinical data of pregnant women to identify risk factors for adverse outcomes. This study aimed to investigate the correlation between the FFA profile in the second trimester and APO in pregnant women with GDM and identify the ideal cutoff value for predicting adverse maternal and infant outcomes.

## 2 Materials and methods

### 2.1 Study population

The study included pregnant women who visited Shengjing Hospital affiliated to China Medical University between December 2022 and December 2023. The inclusion criteria were as follows (1): patients who met the diagnostic criteria for GDM, (2) not < 20 years, (3) and those having single pregnancy. The exclusion criteria were as follows: (1) pregnant women with pre-GDM (PGDM) who underwent an oral glucose tolerance test (OGTT) before or during the first trimester, (2) Incomplete clinical data, (3) History of autoimmune diseases, tumors, severe infections, severe liver and kidney dysfunction, hematological diseases, or GDM, (4) Smoking or alcohol consumption. Finally, 196 pregnant women were admitted to the study as the final analytic population and included 52 controls, 144 GDM cases ( 83 normal pregnancy outcome (NPO) cases, 61 APO cases ). Ethical approval for this study was obtained from the Medical Ethics Committee of Shengjing Hospital of China Medical University (ethics number: 2023PS809k).

*Diagnostic criteria:*

GDM was defined as either a fasting plasma glucose (FPG) > 5.1 mmol/L, OGTT 1-h plasma glucose(OGTT1 h PG) > 10.0 mmol/L, or OGTT 2-h plasma glucose(OGTT2 h PG) > 8.5 mmol/L (30).

APOs is defined as any combination of the following adverse maternal and infant outcomes: maternal outcomes, including HDCP (hypertension during pregnancy, preeclampsia) and emergency cesarean section. Neonatal outcomes included large for gestational age (LGA), small for gestational age (SGA), macrosomia, LBW, preterm birth, and stillbirth. Preterm birth was defined as live birth with a gestational age > 28 weeks and < 37 weeks, and stillbirth was defined as fetal death *in utero* at 20 weeks of gestation. LGA was defined as the 90th percentile of fetal birth weight greater than the normal weight for gestational age, SGA was defined as fetal birth weight less than the 10th percentile of normal weight for gestational age, macrosomia was defined as birth weight > 4,000 g, and LBW was defined as birth weight < 2,500 g. Gestational hypertension was defined as blood pressure > 140/90 mmHg after 20 weeks of pregnancy, and preeclampsia was defined as blood pressure > 140/90 mmHg and proteinuria > 300 mg/day after 20 weeks of pregnancy. Emergency cesarean section is defined as an emergency operation that seriously threatens the life of the mother and child, while NPO was defined as the absence of these adverse outcomes.

### 2.2 Data and sample collection

General clinical data and laboratory examination indicators were collected during pregnancy, and it mainly included general information and indicators of mothers, such as age, gestational age, pre-pregnancy body mass index (pBMI), gestational weight gain (GWG), systolic blood pressure, diastolic blood pressure (DBP), gravidity, parity, pre-pregnancy hypertension, assisted reproduction, blood glucose control method, adverse pregnancy history(preterm birth, miscarriage, stillbirth, etc.), and hypothyroidism (decreased production or action of thyroid hormones, resulting in systemic hypometabolism syndrome). A 2-h 75- g OGTT was performed for all participants at 24–28 weeks gestation, and, at the same time, biochemical laboratory indicators were also tested. Fetal indicators included fetal sex and birth weight.

### 2.3 Measurements

#### 2.3.1 Material

FPG, OGTT1 h PG, and OGTT2 h PG were measured using a glucose assay kit (hexokinase method), and glycated hemoglobin (HbA1c) was separated using high-pressure liquid ion exchange chromatography. TG was measured using a TG assay kit (GPO-PAP method), total cholesterol(TC) was measured using a total cholesterol assay kit (CHOD-PAP method), High density lipoprotein cholesterol(HDL-cholesterol) was measured using a high-density lipoprotein cholesterol assay kit (direct method-catalase clearance method), and Low density lipoprotein cholesterol(LDL-cholesterol) was measured using a low-density lipoprotein cholesterol assay kit (direct method-catalase clearance method).

Serum FFA profiles detection with liquid chromatography-mass spectrometry (LC-MS). AB SCIEX Triple Quad™ 4500MD LC-MS /MS system (ABsciex, Toronto, Canada) was used for sample analysis. The column used was the ACQUITY UPLC BEH C18 1.7um, WATERS.

#### 2.3.2 Serum fatty acids

Serum pretreatment :fasting venous blood samples were collected from pregnant women at 24–28 weeks of gestation, placed in anticoagulant-free collection vessels, centrifuged at low temperature (4°C, 12,000 r/ min for 10 min), and the supernatant was separated into EP tubes and stored in a refrigerator at –80°C. Serum samples (5 μL) were carefully aspirated and placed in a clean glass tube. We added 50 μL of internal standard solution and 1,000 μL of dissociation solution, shook at 1,850 rpm for 10 s, heated at 80°C for 20 min in a constant temperature mixer, added 80 μL of neutralization solution and 1 mL of extractant, shook at 1,850 rpm for 5 min, and let it rest in the hood for 5 min. We blow-dried 700 μL of supernatant at 50°C under nitrogen for 5 min. Then, 400 μL of the mixture of methanol and acetonitrile was added and shaken at 1,850 rpm for 5 min. We put 100 μL into a 96-well plate and injected it for analysis.

Serum FFA profiles were measured using LC-MS. FFA levels in serum were expressed as the absolute concentration of fatty acids (μmol/L). Twenty-four FFA types were detected, as follows: total FFA, SFA, MUFA, PUFA, n–6 PUFA, and n–3 PUFA. Total SFA (palmitic [C16:0], stearic [C18:0], arachidic [C20:0], behenic acid [C22:0], lignoceric acid [C24:0]), MUFA (palmitoleic acid [C16:1], oleic acid [C18:1], eicosenoic acid [C20:1], nervonic acid [NA,C24:1]), PUFA (Total n–3 PUFA (alpha-linolenic acid, [ALA, C18:3], eicosapentaenoic acid [EPA, C20:5], docosapentaenoic acid [DPA, C22:5], docosahexaenoic acid [DHA, C22:6]), and Total n–6 PUFA (linoleic acid [LA, C18:2], eicosadienoic acid [C20:2], scolic acid [C20:3], arachidonic acid [AA, C20:4], eicosatetraenoic acid [C22:4]) ).

### 2.4 Statistical analysis

In the general data analysis, continuous variables were tested for normality, and fitted normal distributions were compared using Student’s t test and expressed as mean ± standard deviation (SD). Non-conforming normal distributions were compared using non-parametric tests and represented by medians and quartiles. Categorical variables were compared using the chi-squared test and expressed as numbers and percentiles. Multivariate logistic regression was used to assess the relationship between the FFA profile and APO. Receiver operating characteristic (ROC) curve was used to evaluate the predictive value of the FFA spectrum for APO in women with GDM. IBM® SPSS@Statistics v27.0 software was used for data collating and statistical analysis. Statistical significance was set at p < 0.05.

## 3 Results

### 3.1 General characteristics of the participants

A total of 196 pregnant women at 24−28 weeks of gestation who met the inclusion criteria were included in the study. **Tables 1**, **2** shows the basic clinical characteristics of pregnant women. Adverse pregnancy history, FPG, OGTT1hPG, OGTT2hPG, 25-hydroxyvitamin D3, birth weight, and fetal sex differed between the NPO group and the NC group. Differences were observed in gestational age, pBMI, GWG, FPG, OGTT1hPG, OGTT2hPG, HbA1c, and ferritin levels between the APO group and the NC group. Significant differences were observed in gestational age, pBMI, DBP, FPG, ferritin, and fetal birth weight between the NPO and APO groups (P < 0.05); The number and proportion of APO are shown in **Table 3**.

**Table 1:** Demographic characteristics of participants at 24 – 28 gestational weeks.

Variable	NC (n=52)	NPO (n=83)	APO (n=61)
Mother			
Age, year	31.04 ± 3.55	31.85 ± 4.12	32.42 ± 4.49
Gestational age, week	38 (38,39)	38 (38,39)	37 (35,39)^#†^
pBMI, kg/m^2^	22.66 ± 4.53	23.85 ± 3.91	26.05 ± 4.28^#†^
GWG, kg	15.00 (11.00,19.75)	12.00 (9.20,16.25)	10.50 (7.50,13.25)^#^
SBP, mmHg	120.00 (110.00,130.00)	120.00 (115.00,125.00)	120.50 (118.00,129.50)
DBP, mmHg	78.38 ± 10.76	75.79 ± 7.85	80.22 ± 12.09^†^
Gravidity	1.50 (1.00,2.00)	2 (1,3)	2 (1,2)
Parity	1.00 (1.00,2.00)	1 (1,2)	1 (1,1)
Therapy			
Diet (%)	/	73 (88.0)	55 (90.2)
Medicine (%)	/	10 (12.0)	6 (9.8)
Adverse pregnancy history		^*^	
No (%)	38 (79.2)	49 (59.0)	32 (52.5)
Yes (%)	10 (20.8)	34 (41.0)	29 (47.5)
Hypothyroidism			
No (%)	41 (85.4)	73 (88.0)	54 (88.5)
Yes (%)	7 (14.6)	10 (12.0)	7 (11.5)
Pre-pregnancy hypertension			
No (%)	46 (95.8)	81 (97.6)	57 (93.4)
Yes (%)	2 (4.2)	2 (2.4)	4 (6.6)
Assisted reproduction			
No (%)	46 (95.8)	76 (91.6)	54 (88.5)
Yes (%)	2 (4.2)	7 (8.4)	7 (11.5)
Fetal			
Birth weight, kg	3154.60 ± 458.54	3299.27 ± 301.88^*^	3013.36 ± 683.36^†^
Fetal sex		^*^	
Male, n (%)	29 (61.7)	34 (41.0)	29 (47.5)
Female, n (%)	18 (38.3)	49 (59.0)	32 (52.5)

Data are given as a number (percentage) for categorical variables and (mean ± SD) or median (IQR) for continuous variables. ^*^p < 0.05 NC vs. NPO; ^#^p < 0.05 NC vs. APO; †p < 0.05 NPO vs. APO. pBMI, pre-pregnancy body mass index; GWG, gestational weight gain; SBP, systolic pressure; DBP, diastolic blood pressure.

**Table 2:** Biochemical laboratory index of participants at 24 – 28 gestational weeks.

Variable	NC (n=52)	NPO (n=83)	APO (n=61)
FPG, mmol/L	4.61 ± 0.25	5.05 ± 0.49^*^	5.22 ± 0.40^#†^
OGTT1hPG,mmol/L	7.72 ± 1.35	9.75 ± 1.35^*^	10.05 ± 1.42^#^
OGTT2hPG,mmol/L	6.73 ± 0.88	8.04 ± 1.33^*^	8.36 ± 1.22^#^
HbA1c,%	4.89 ± 0.27	5.08 ± 0.70	5.17 ± 0.32^#^
TG, mmol/L	4.55 ± 2.62	2.63 ± 1.20	2.87 ± 1.28
TC, mmol/L	3.55 ± 1.52	5.19 ± 1.40	4.92 ± 1.13
HDL, mmol/L	1.99 ± 0.64	1.69 ± 0.44	1.57 ± 0.37
LDL, mmol/L	3.13 ± 1.00	2.80 ± 0.99	2.65 ± 0.75
Ferritin, ng/mL	10.05(6.23,19.83)	10.80(8.30,17.25)	24.40(10.55,34.55)^#†^
25-hydroxyvitamin D3, ng/mL	20.48 ± 7.08	24.49 ± 6.77^*^	24.01 ± 8.78

Data are given as (mean ± SD) or median (IQR) for continuous variables. ^*^p < 0.05 NC vs. NPO; ^#^p < 0.05 NC vs. APO; ^†^p < 0.05 NPO vs. APO. FPG, Fasting plasma glucose; OGTT1hPG, 1-h plasma glucose; OGTT2hPG, 2-h plasma glucose; HbA1C, Glycosylated hemoglobin; TG, Triglyceride; TC, Total cholesterol; HDL, High density lipoprotein cholesterol; LDL, Low density lipoprotein cholesterol.

**Table 3:** Classification of adverse pregnancy outcomes.

Pregnancy outcome	Classify	N (%)
APO (n=61)	LGA	10 (16.4)
	SGA	15 (24.6)
	Macrosomia	7 (11.5)
	LBW	15 (24.6)
	Preterm birth	21 (34.4)
	Stillbirth	1 (1.6)
	Emergency Cesarean section	13 (21.3)
	HDCP	32 (52.5)
	Oligoamnios	2 (3.3)

LGA, Large for gestational age; SGA, Small for gestational age; LBW, low birth weight; HDCP, Hypertensive disorder complicating pregnancy.

### 3.2 Differences in FFA profiles between 24 and 28 weeks in pregnant women

Differences in the FFA spectrum levels measured between the three groups of pregnant women at 24–28 weeks were compared, and the results are shown in **Table 4**. Differences were observed in total n-3 and DHA between the NPO and APO groups and the NC group. Additionally, C20:0 and the ratio of total n-3 to n-6 differed between the NC and APO groups.

**Table 4:** Differences in FFA profiles between participants at 24−28 weeks of gestation.

FFAs (μmol/L)	NC (n=52)	NPO (n=83)	APO (n=61)
Total FFA	28468.24 (23296.33,31390.01)	29499.24 (24059.90,33707.17)	27368.79 (22805.10,33117.34)
Total SFA	9419.68 (7645.26,10351.29)	9368.98 (7749.79,11251.52)	7958.49 (6917.58,10426.88)
C16:0	6970.82 (5575.47,7733.56)	6991.05 (5862.94,8406.87)	6251.25 (5205.23,7778.74)
C18:0	1980.10 ± 515.06	2129.85 ± 628.00	1818.52 ± 560.17^†^
C20:0	65.84 ± 18.49	63.14 ± 17.52	58.07 ± 19.04^#^
C22:0	58.19 (44.71,77.47)	58.55 (43.17,74.23)	59.40 (41.98,70.21)
C24:0	63.63 (48.90,76.51)	62.90 (51.92,80.96)	62.56 (50.83,76.12)
Total MUFA	3476.93 (2628.91,4515.01)	3762.56 (2864.29,4864.70)	3666.96 (2691.71,4589.48)
C16:1	256.27 (179.83,392.52)	300.00 (203.32,377.08)	280.85 (188.80,429.13)
C18:1	3011.42 (2377.62,3842.76)	3331.28 (2490.97,4208.14)	3283.63 (2371.01,3990.60)
C20:1	25.34 (19.00,28.74)	27.94 (19.33,38.59)	23.81 (18.32,33.05)
C24:1	98.52 (81.12,118.51)	100.71 (84.24,118.09)	92.99 (73.90,117.71)
Total PUFA	15106.57 ± 2955.84	16171.17 ± 3976.28	15703.81 ± 3120.35
Total n-3	1409.43 (1144.20,1710.37)	1558.32 (1335.85,1997.25)^*^	1579.35 (1267.08,2033.21)^#^
C18:3,ALA	218.18 (172.32,270.46)	228.57 (173.80,281.78)	216.74 (185.27,307.73)
C20:5,EPA	94.32 (71.45,132.69)	104.22 (80.78,150.42)	107.70 (89.24,134.08)
C22:5,DPA	235.39 (197.12,340.03)	260.32 (207.88,316.76)	245.27 (190.00,332.62)
C22:6,DHA	1094.69 (891.99,1359.85)	1199.82 (1029.16,1629.87)^*^	1237.57 (981.81,1680.19)^#^
Total n-6	12801.42 ± 2408.54	13643.61 ± 3313.76	13142.11 ± 2513.34
C18:2,LA	9045.86 ± 1765.42	9500.22 ± 2303.69	9052.14 ± 1728.48
C20:2	64.10 (55.41,77.46)	69.71 (51.81,89.19)	65.13 (51.79,80.69)
C20:3	392.81 ± 128.85	394.55 ± 142.49	413.65 ± 122.19
C20:4,AA	3692.23 (2983.52,4558.55)	3891.31 (3273.74,4912.14)	3961.27 (3265.10,4833.68)
C22:4	78.91 (62.50,99.42)	80.91 (59.58,99.00)	79.21 (60.04,103.69)
Total n-3/n-6	0.12 ± 0.03	0.13 ± 0.03	0.13 ± 0.03^#^

^*^p < 0.05 NC vs. NPO; ^#^p < 0.05 NC vs. APO; ^†^p < 0.05 NPO vs. APO. AA, arachidonic acid; ALA, alpha-linolenic acid; DHA, docosahexaenoic acid; DPA, docosapentaenoic acid; EPA, eicosapentaenoic acid; LA, linoleic acid; MUFAs, monounsaturated fatty acids; PUFAs, polyunsaturated fatty acids; SFAs, saturated fatty acids; FFA, free fatty acid.

The C18:0 level in the Total SFA showed a significant difference between the NPO and APO groups (p<0.05), Upon further stratification by fetal sex, differences in C18:0 were observed in female fetuses (**Table 5**).

**Table 5:** Differences in FFA profiles of subjects stratified by fetal sex between 24−28 weeks of gestation.

FFAs (μmol/L)	Male (n=63)		Female (n=81)	
	NPO (n=34)	APO (n=29)	NPO (n=49)	APO (n=32)
Total FFA	29934.49 ± 8903.03	28156.02 ± 6757.16	29628.75 ± 6546.93	28603.44 ± 6356.23
Total SFA	9580.05 ± 2942.66	8847.57 ± 2620.36	9668.00 ± 2462.48	8821.94 ± 2493.08
Total MUFA	4037.70 ± 1747.51	3759.67 ± 1446.25	3885.52 ± 1208.04	3943.34 ± 1367.11
Total PUFA	16316.73 ± 4621.35	15548.78 ± 3223.26	16075.23 ± 3541.67	15838.17 ± 3077.23
Total n-3/n-6	0.12 ± 0.04	0.13 ± 0.03	0.13 ± 0.03	0.13 ± 0.04
Total n-3	1675.75 ± 672.62	1711.73 ± 501.57	1584.88 (1394.36,1959.28)	1492.25 (1287.36,2006.73)
C20:0	61.91 ± 19.68	58.92 ± 15.86	63.95 ± 16.13	57.34 ± 21.66
C22:6,DHA	1153.73 (1005.00,1762.20)	1359.66 (1108.78,1703.26)	1257.04 (1036.63,1617.52)	1194.90 (970.35,1648.97)
C18:0	2001.44 (1781.98,2491.95)	1740.74 (1495.59,2174.70)	2127.92 ± 618.13	1806.74 ± 581.44^†^

^†^p < 0.05 NPO vs. APO. DHA, docosahexaenoic acid; MUFAs, monounsaturated fatty acids; PUFAs, polyunsaturated fatty acids; SFAs, saturated fatty acids; FFA, free fatty acid.

### 3.3 Regression analysis of APO occurrence in pregnant women with GDM

According to the level of C18:0 from low to high, we divided C18:0 levels into three groups according to the tertile (T): T1, T2, and T3. The C18:0 level of the T1 group was the lowest, and that of the T3 group was the highest. Groups T2 and T3 correlated with APO (OR= 0.298, 95%CI: 0.128–0.690) and (OR= 0.359, 95%CI: 0.157–0.822), respectively. After adjusting for pBMI, FPG, DBP, and ferritin, Fetal sex, group T3 showed an independent correlation with APO (OR= 0.186, 95%CI: 0.047–0.736) (**Table 6**).

**Table 6:** Logistic regression analysis of C18:0 and the occurrence of APO in patients with GDM.

	T1 (n=48)	T2 (n=48)	*P*	T3 (n=48)	*P*
Model 1	1.000 (reference)	*0.298 (0.128,0.690)*	*0.005*	*0.359 (0.157,0.822)*	*0.015*
Model 2	1.000 (reference)	0.428 (0.170,1.080)	0.072	*0.370 (0.149,0.922)*	*0.033*
Model 3	1.000 (reference)	*0.379 (0.146,0.982)*	*0.046*	*0.342 (0.134,0.871)*	*0.025*
Model 4	1.000 (reference)	*0.314 (0.112,0.883)*	*0.028*	*0.362 (0.134,0.979)*	*0.045*
Model 5	1.000 (reference)	0.345 (0.085,1.407)	0.138	*0.186 (0.047,0.736)*	*0.017*

Italic values indicate a significant p-value (p < 0.05). CI, confidence interval; OR, odds ratio. Model 1: unadjusted odds ratio (OR); Model 2: adjusted for pBMI; Model 3: adjusted for pBMI, FPG; Model 4: adjusted for pBMI, FPG, DBP; Model 5: adjusted for pBMI, FPG, DBP, ferritin, Fetal sex.

### 3.4 Ability of univariate and multivariate ROC analysis C18:0 and general clinical data to screen for APO occurrence in pregnant women with GDM

#### 3.4.1 C18:0 ability to predict APO alone and with general clinical data

**Figure 1:**
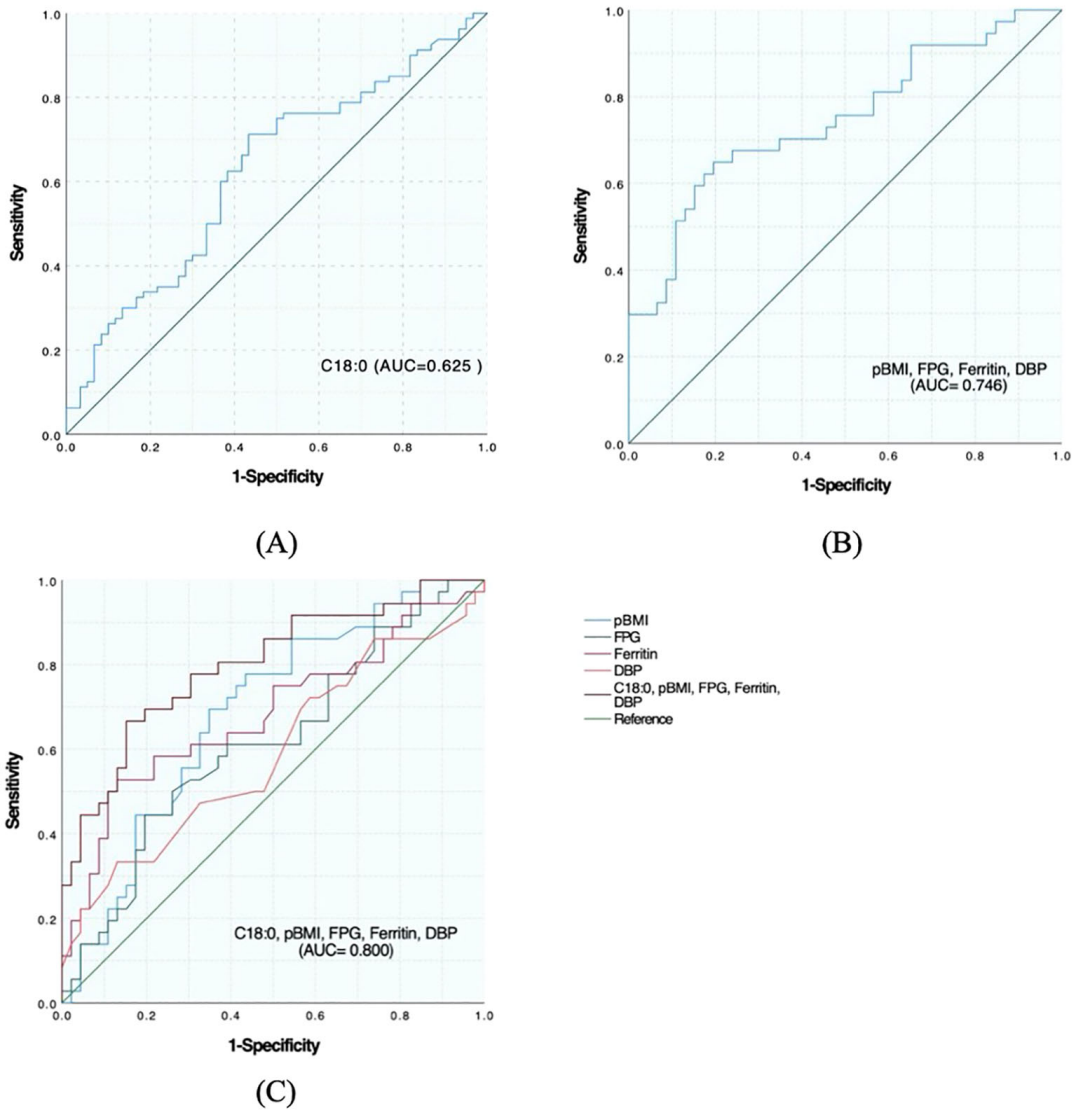
Predictive effect of C18:0 level and its combination with other indicators on the occurrence of APO in pregnant women with GDM. **(A)** ROC analysis of C18:0. **(B)** ROC analysis after combination of pBMI, FPG, Ferritin and DBP. **(C)** ROC analysis after C18:0 combined with pBMI, FPG, Ferritin, and DBP.

The area under the curve (AUC) of significant FFA (C18:0) for screening APO was 0.625 (95%CI: 0.531–0.718), indicating a certain predictive ability. According to the maximum value of the Youden index, the screening cutoff value of screening was C18:0 1776.68 μmol/L. In general, for clinical data, the AUC of pBMI was 0.686 (95%CI: 0.571–0.801), AUC of FPG was 0.615 (95%CI: 0.491–0.738), AUC of ferritin was 0.684 (95%CI: 0.563–0.805), and AUC of DBP was 0.590 (95%CI: 0.463–0.717). The AUC of pBMI, FPG, ferritin and DBP combined for screening APO was 0.746 (95%CI: 0.638-0.855). The AUC of C18:0 with general clinical data for screening APO was 0.800 (95%CI: 0.702–0.897), which was improved compared with that of screening alone (**Figures 1A–C**).

#### 3.4.2 Ability of C18:0 alone and with general clinical data to predict each adverse outcome

**Figure 2:**
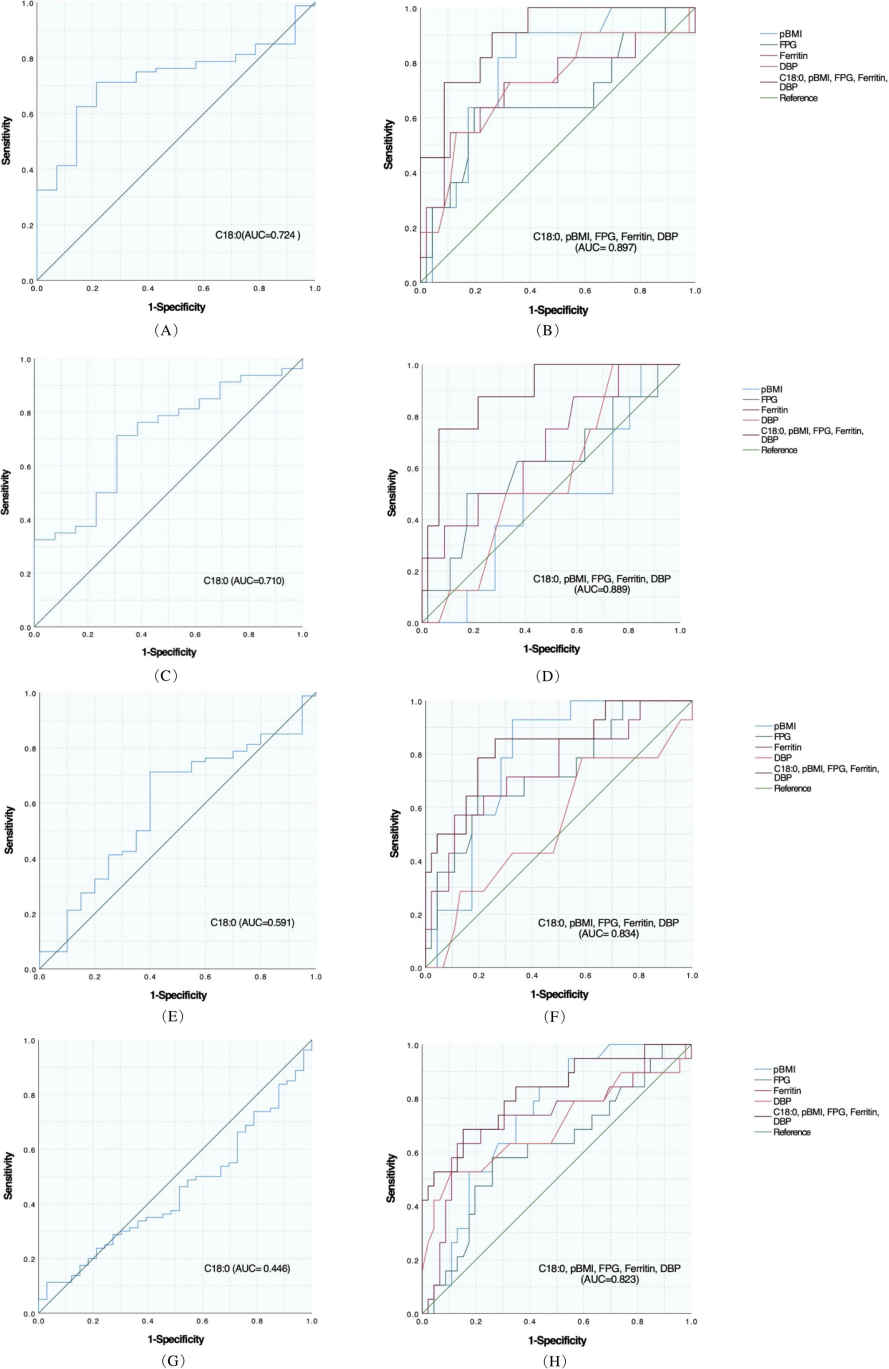
Predictive effect of C18:0 level and combined general information on each adverse outcome in women with GDM. **(A)** ROC analysis of C18:0 for LBW. **(B)** ROC analysis of C18:0 combined with pBMI, FPG, Ferritin, and DBP for LBW. **(C)** ROC analysis of C18:0 for Emergency cesarean section. **(D)** ROC analysis of C18:0 combined with pBMI, FPG, Ferritin, and DBP in Emergency cesarean section. **(E)** ROC analysis of C18:0 for Preterm birth. **(F)** ROC analysis of C18:0 combined with pBMI, FPG, Ferritin, and DBP for Preterm birth. **(G)** ROC analysis of C18:0 for HDCP. **(H)** ROC analysis of C18:0 combined with pBMI, FPG, Ferritin, and DBP for HDCP.

ROC curve was used to evaluate the identification and prediction ability of C18:0 for infants with LBW, emergency cesarean section, premature delivery, and HDCP, and the results are shown in **Table 7**, **Figures 2A, C, E, G**. For the prediction and diagnosis of infants with LBW, the optimal C18:0 cutoff value was 1761.36 μmol/L, with a sensitivity of 71.3% and specificity of 78.6% (AUC=0.724, 95%CI: 0.609–0.839). For the diagnosis of emergency cesarean section, the optimal C18:0 cutoff value was 1761.36 μmol/L, with a sensitivity of 71.3% and specificity of 69.2% (AUC= 0.710, 95%CI: 0.569–0.850). For the prediction and diagnosis of preterm birth, the optimal C18:0 cutoff value was 1776.68 μmol/L, with a sensitivity of 71.3% and specificity of 60.0% (AUC =0.591, 95%CI: 0.453–0.729). For the prediction and diagnosis of HDCP, the optimal C18:0 cutoff value was 569.66 μmol/L, with a sensitivity of 11.3% and specificity of 97.0 % (AUC= 0.446, 95%CI: 0.334–0.557). We further compared the ability of C18:0, pBMI, FPG, DBP, and ferritin binding to identify and predict infants with LBW, emergency cesarean section, premature delivery, and HDCP, and the results are shown in **Figures 2B, D, F, H**. The ability of C18:0 with general clinical data to identify and predict LBW (AUC =0.897, 95%CI: 0.807–0.988) and emergency cesarean section (AUC =0.889, 95%CI: 0.777–1.000), preterm birth (AUC= 0.834, 95%CI: 0.708–0.960), and HDCP (AUC= 0.823, 95%CI: 0.705–0.940) was better than that of C18:0 alone.

**Table 7:** ROC curves to evaluate the predictive power of C18:0 for adverse outcomes.

Outcome	AUC	95%Cl	Sensitivity	1– Specificity
LBW	0.724	0.609-0.839	0.713	0.214
Emergency cesarean section	0.710	0.569-0.850	0.713	0.308
Preterm birth	0.591	0.453-0.729	0.713	0.400
HDCP	0.446	0.334-0.557	0.113	0.030

LBW, low birth weight; HDCP, Hypertensive disorder complicating pregnancy; AUC, Area under the curve; CI, confidence interval.

## 4 Discussion

In this study, the serum pBMI levels in patients with GDM were observed to be higher compared to those in the control group. Moreover, this disparity was found to be statistically significant within the APO group. This is consistent with the findings of Wang et al., who reported a significantly increased risk of GDM associated with elevated pBMI (31). Pregnant women with GDM can experience complications with various APO and GDM is related to long-term childhood obesity and abnormal glucose tolerance (17, 18). The results of this study revealed that the APO group presented a higher pBMI compared to the healthy control group and the NPO group, and a lower GWG in comparison to the healthy control group. Extant literature has demonstrated that an elevated pBMI is associated with an increased risk of adverse outcomes in GDM, and that inadequate GWG is linked to a higher risk of preterm birth, a pattern that is echoed in the findings of this study (32). Another study suggested that FPG at 24−30 gestational weeks was closely related to APO in women with GDM (33). Higher serum ferritin levels in the second trimester are significantly associated with the risk of GDM and GDM-related APO, especially preeclampsia (34). HbA1c are also associated with the occurrence of APO (35). In this study, fasting blood glucose levels and ferritin levels in the APO group exceeded those in both the control group and the NPO group, HbA1c was greater than the control group. However, recent research has indicated a potential association between lipid levels and the incidence of GDM and APO (36, 37), In the present study, lipid levels exhibited no significant differences across the three cohorts.

The FFA profile contains many specific FFA. In the past five years, studies on FFA profiles have gradually increased. In normal early pregnancy, maternal obesity promotes IR and leads to increased lipolysis in the second trimester (5, 38). Elevated maternal IR leads to elevated maternal postprandial blood glucose levels and growth-promoting FFAs (3, 38, 39). Meanwhile, the FFA level/profile is closely related to IR, and circulating FFA is an important factor that promotes IR and alters insulin secretion (40, 41). GDM FFA levels are significantly elevated in women with GDM (24). FFA profiles may be involved in GDM development, In early pregnancy, Ma et al. and Zhang et al. reported elevated levels of myristic and palmitic acids in pregnant women with GDM. Ma et al. found a positive association between palmitic acid and GDM risk. In our previous review (42), Total plasma SFA, MUFA, PUFA n-6, and PUFA n-3 levels increased in pregnant women with GDM in early pregnancy (43), In a study by Zhang et al., dietary supplementation with ALA and DHA was associated with the risk of GDM (11, 13). It found that supplementation with DHA, sourced from fish oil, did not demonstrate efficacy in preventing the onset of GDM (44), whereas the consumption of n-3 PUFA among pregnant women diagnosed with GDM was associated with a potential alleviation of IR and inflammation, as well as a reduced risk of adverse pregnancy outcomes (45, 46). In our study, the analysis of the FFA profile revealed that DHA and PUFA n-3 levels were elevated in the GDM group relative to the healthy control group, and were the most pronounced in the APO group. However, there was no significant difference between the NPO and APO groups.

Maternal plasma lipids during pregnancy could promote intrauterine fetal growth through the stimulation of placental insulin-like growth factor-1 (IGF-1) secretion. Chen et al. suggest that maternal cord blood IGF-1 concentrations were higher in fetuses delivered LGA, Maternal plasma levels of free C16:0 and C18:0 were significantly associated with cord blood IGF-1 concentrations. Treatment with C16:0, C18:0, and C18:2 could induce the expression and secretion of IGF-1 in human trophoblast 3A-sub E cells (47). It was observed in this study that the concentration of C18:0 varied between the NPO and APO groups, with lower levels observed in the APO group. Furthermore, fetal birth weight was found to be reduced in the APO group, and logistic regression analyses revealed an inverse association between low、moderate levels of C18:0 and the incidence of APO. However, the association of C18:0 with fetal growth and development requires further investigation to elucidate the underlying mechanisms.

Presently, few studies have analyzed FFA profiles and pregnancy outcomes in GDM. Higher FFA levels can lead to an increase in fetal birth weight (24), and maternal FFA levels are positively correlated with the prevalence of LGA. In addition, high 2h-FFA levels in the second trimester, but not fasting FFA levels, are associated with an increased risk of delivering LGA neonates (25, 26). The study by Herrera et al. showed that in pregnant women with GDM with good glycemic control, maternal FFA positively correlated with neonatal weight and fat mass, which may be because dyslipidemia in mothers with GDM promoted the transfer of maternal fatty acids to the fetus, increasing fetal fat tissue mass, thereby increasing the risk of macrosomia (48). However, in the study by Fan et al., FFA levels of women with GDM complicated with FGR in the third trimester were higher than that of the control group, and the AUC value of diagnosing GDM complicated with FGR was 0.84 (27). We concluded that the C18:0 level in the FFA spectrum has a certain predictive value and can predict the occurrence of LBW, emergency cesarean section, and premature delivery in women with GDM. Considering the multitude of factors influencing APO in pregnant women with GDM, the integration of C18:0 levels with general clinical data enhances the predictive capacity for APO. The AUC values for predicting LBW and emergency cesarean section in women with GDM were >0.85, and the AUC values for predicting preterm birth and HDCP were >0.80. C18:0 should be included when considering the occurrence of APO. However, the enrolled subjects do not represent a random sample of Chinese pregnant women. Furthermore, an analysis determining the FFA profile throughout various stages of pregnancy is absent, and additional experimental studies, including animal models and prospective cohort analyses, are required to elucidate the mechanisms by which the FFA profile is associated with APO. The findings of this study offer valuable insights for the development of effective disease prevention strategies and the establishment of prompt maternal and neonatal assessment protocols.

## 5 Conclusion

At present, most studies have analyzed the correlation between FFA levels and APO in pregnant women and have suggested that it has a certain research value. Our study found that the C18:0 level detected at 24–28 weeks of gestation was independently related to APO in pregnant women with GDM and had predictive efficacy. C18:0 combined with general clinical data (pBMI, FPG, DBP, and ferritin) improved the predictive power. Similar results were obtained while studying LBW, emergency cesarean section, premature delivery, and HDCP in pregnant women with GDM. In the future, multicenter studies with large sample sizes are needed to analyze the influence of FFA spectrum levels at different pregnancy periods on APO in pregnant women with GDM.

## Data Availability

The raw data supporting the conclusions of this article will be made available by the authors, without undue reservation.
